# Supplementary material for: Seedling Characteristics of Three Oily Species before and after Root Pruning and Transplant
Source: Plants (Basel). 2019 Jul 30;8(8):258. doi: 10.3390/plants8080258 (PMC6724410; doi:10.3390/plants8080258)
Supplement: Supplementary File 1 [file plants-08-00258-s001.zip › Supplementary.docx]

**S1.** Average ±SE leaf dimensions and weights of 63 day-old *Jatropha*, *Moringa* and *Ricinus* seedlings.

| **Parameter** | **Moringa** | **Ricinus** | **Jatropha** |
| --- | --- | --- | --- |
| Blade thickness without veins (mm) | 0.23±0.01**b** | 0.24±0.01**b** | 0.26±0.01**a** |
| Blade thickness including veins (mm) | 0.16±0.01**b** | 0.29±0.02**a** | 0.35±0.01**a** |
| Length of blade (mm) | 22.00±1.43**c** | 228.17±9.67**a** | 136.17±1.36**b** |
| Width of blade (mm) | 14.08±0.71**c** | 194.83±11.41**a** | 117.33±2.93**b** |
| Weight of one blade (mg) | 32.48±2.60**c** | 4111.80±401.10a | 3214.30±46.41**b** |
| Thickness of petiole at the base of the blade | 0.52±0.02**b** | 3.49±0.20**a** | 3.33±0.05**a** |
| Thickness of petiole at the base of the bud | 0.69±0.04**b** | 4.72±0.4 **a** | 4.24±0.13**a** |
| Length of petiole | 42.33±3.22**c** | 87.50±4.29**a** | 68.33±2.04**b** |
| Weight of petiole (mg) | 12.12±0.98**c** | 1181.20±169.10**a** | 666.95±26.78**b** |

For Moringa only one leaflet of the compound leaf was selected. Different letters in the same row indicate statistical differences (P<0.05).





S2. Root diameter distributions along vertical and horizontal axes of *Jatropha*, *Moringa* and *Ricinus*, 83 days post transplanting.
